# Case Report: Uncommon complications of central catheters in newborns: two cases of parenteral nutrition extravasation

**DOI:** 10.3389/fped.2025.1602098

**Published:** 2025-06-05

**Authors:** Carolina Coramusi, Jessica F. Toro, Ramón G. Pabón, Mario Barreto, Giovanni Di Nardo, Pasquale Parisi, Claudia C. M. Marín

**Affiliations:** ^1^ Unit of Pediatrics, Sant'Andrea University Hospital, Faculty of Medicine and Psychology, “Sapienza” University, Rome, Italy; ^2^ Clinica Medilaser Abner Lozano, Pediatric Infection Diseases, Faculty of Medicine UniNavarra University Foundation, Neiva, Colombia; ^3^ Clinica Medilaser Abner Lozano, Neonatal Intensive Care Unit, Faculty of Medicine, UniNavarra University Foundation, Neiva, Colombia

**Keywords:** newborn, umbilical venous catheter, Epicutaneo-Caval Catheter, complications, cystic lesions, hydrothorax, pneumothorax, case report

## Abstract

Central venous catheters (CVCs) are essential in neonatal care units to ensure prolonged venous access. Despite experienced CVC placement, managing fragile and small newborn vessels involves the risk of traumatic and iatrogenic complications. The most common include phlebitis, thrombosis, sepsis, and catheter displacement. Rarely, catheter displacement may lead to fluid accumulation around serous membranes and adjacent organs, which, if not identified early, can even result in neonatal death. We report two cases: one of neonatal hydrothorax with subsequent lung cystic formation after a parenteral nutrition leakage caused by the displacement of an Epicutaneo-Caval Catheter in the basilic vein of the left arm; the second involves a peritoneal collection of parenteral nutrition from the umbilical venous catheter.

## Introduction

Advancements in neonatal intensive care have improved survival rates for VLBWN with serious pathologies or complex malformations. This improvement has led to extended hospital stays to provide complex therapies. Consequently, various techniques aim to enhance the quality and safety of prolonged venous access.

Central venous catheters (CVCs) are crucial for managing neonates in intensive care units. These devices are indispensable for administering lifesaving medications, parenteral nutrition (PN), and monitoring central pressures (1, 2). However, their use in neonatal intensive care settings presents challenges, especially in small preterm newborns, whose fragile blood vessels increase the risk of complications (2). Recently, the placement of silicone catheters through a peripheral vein, known as Epicutaneo-Caval Catheter (ECC), has become the preferred approach (3).

ECCs are employed for newborns requiring intravenous treatments for periods longer than 7 days; positioning is relatively simple and can be performed at the patient's bedside (3, 4). Advantages include less trauma during installation, increased duration, and reliability of access. Indwelling ECCs are not exempted from complications, e.g., thrombosis, phlebitis, and liquid infiltration/extravasation. The most severe include catheter-related bloodstream infections (CLABSI), arrhythmias, catheter displacement with pleural and pericardial effusion, or pericardial tamponade (3, 5).

Therefore, to minimize complications when using a CVC in a neonate, it is essential to carefully assess the potential benefits against the risks and adhere to stringent protocols for insertion and maintenance (5, 6). In this context, we discuss a rare complication associated with CVCs: fluid extravasation due to tip migration.

Specifically, we present two cases: one involving a 30-week gestational age neonate who developed lung cysts following PN extravasation from an ECC displacement into the pleura, and another case in which a term newborn developed an acute abdomen after umbilical venous catheter (UVC) displacement.

## Case 1 report

A female infant was born via vaginal delivery at a thirty-week gestational age (birth weight 1,354 g) to a 24-year-old mother with four previous pregnancies and no risk factors for TORCH infections. The mother has been hospitalized for four days due to a urinary tract infection and preterm premature rupture of membranes (pPROM), 90 h, treated with ampicillin and erythromycin devoid of leucocytosis and increased C-reactive protein (CRP) levels. The amniotic fluid was clear, and the baby adapted well to extrauterine life, with APGAR scores of 8–9–10/10. Oxygen saturation during transport in the incubator remained consistently adequate.

**Figure 1:**
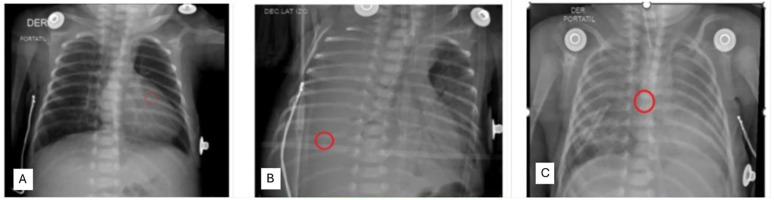
Chest x-ray images: **(A)** tip of the catheter in the right ventricle, with a normal cardiac silhouette. **(B)** Complete opacity of the right hemithorax, indicative of pleural effusion, with hemithorax displacement and tracheal deviation. The over-inserted CVC (approximately 25 cm) is positioned in the right pleural cavity. **(C)** Properly inserted right chest tube with partial resolution of the pleural effusion, a well-positioned endotracheal tube, and an unclearly defined cystic area at the right lung base. (Key features are highlighted with circles).

An indwelling ECC was placed in the basilic vein of the left arm to start first-line antibiotical therapy while awaiting blood culture results to rule out early-onset sepsis; an otherwise normal chest x-ray revealed its intracardial position and needed to be pulled back 3 cm (Figure 1A). Prophylactic antibiotic therapy with ampicillin and amikacin was administered. PN and minimal enteral feeding with breast milk were supplied. Echocardiogram findings excluded pulmonary hypertension. Due to negative blood cultures at 48 h, prophylactic antibiotic therapy was discontinued. The infant then started the kangaroo care protocol and breastfeeding.

On the fourth day of life, the patient's condition deteriorated, with the onset of apnoea episodes, nasal flaring, intercostal retractions, distended abdomen, mottled skin, and jaundice. She was placed on fasting, and new blood cultures and a pneumonia panel were requested. Cefepime was started on suspicion of late-onset neonatal sepsis and pneumonia. Devoid of persistent desaturations, the baby was intubated and placed on high-frequency oscillatory ventilation (HFOV).

The Chest x-ray showed a massive right-sided effusion and mediastinal shift. An over-inserted ECC of about 25 cm was found in the right pleural cavity (Figure 1B). The thoracic surgeon removed 100 cc of milky fluid (PN); the thoracic drainage was secured and the ECC was repositioned. The follow-up chest x-ray showed a partially resolved pleural effusion and a not-well-defined radiolucent cystic area in the right basal field (Figure 1C).

By the fifth day, the patient's clinical condition had improved enough to permit a transition to conventional ventilation. Concurrently, the panel for pneumonia and blood cultures at 48 h was negative; however, Cefepime was continued prophylactically due to the earlier episode of PN extravasation into the pleural cavity. On the sixth day of life, the surgeon clamped the thoracic drain, and the infant was electively extubated, transitioning to high-flow nasal cannula ventilation, which was well-tolerated. PN and fasting were maintained because of the distended and painful abdomen.

**Figure 2:**
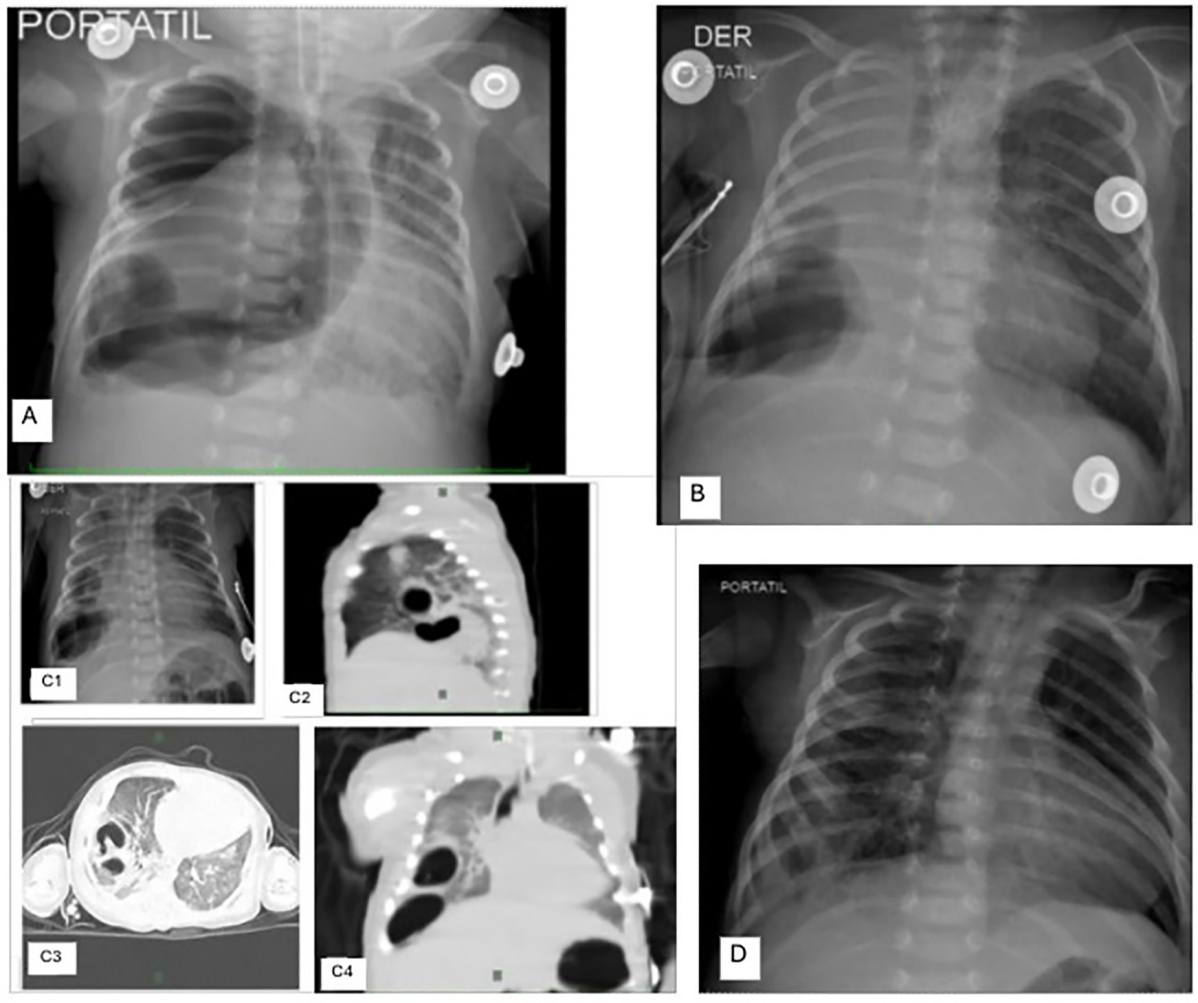
Chest x-ray images: **(A)** right tension pneumothorax with cystic lesions in the right basal lobe and a thoracic tube in place. Note the displacement of the heart and trachea to the left. **(B)** Massive right lung collapse associated with an emphysematous bulla. **(C1–C4)** Chest x-ray and CT scan images: **(C1)** Cystic lesions in the basal right lung; **(C2–C4)** Sagittal, axial, and coronal sections of the chest, highlighting multiple cystic lesions in the right basal area. **(D)** Chest x-ray after 11 days, showing good bilateral lung expansion, homogeneous parenchyma throughout all fields, and a mild increase in peribronchovascular and perihilar interstitial markings.

From the seventh to the fifteenth day, the patient required reintubation twice due to recurrent episodes of respiratory distress associated with a right-sided pneumothorax (Figure 2A), massive right lung collapse, and the development of an emphysematous bulla (Figure 2B), necessitating relocation of the thoracic drain. A follow-up x-ray revealed two well-defined basal right cystic or atelectatic lesions, described on chest CT as “laminar atelectasis and two large bullae in the right hemithorax” (Figure 2C. 1–4). The pediatric surgeon planned a right basal lobectomy for when the infant reached a body weight of at least 2 kg.

The newborn's respiratory status gradually improved; she was extubated on the 22nd day tolerating high-flow nasal cannula ventilation with FiO2 28%. Blood tests from the same day showed a non-infectious picture; antibiotic therapy was suspended on the 23rd day once negative blood cultures were documented.

On the 26th day of life, ventilation was transitioned to low-flow cannulas with minimal oxygen support; in the same day, as the infant tolerated enteral feeding, parenteral nutrition was discontinued.

By the 29th day, a follow-up chest x-ray revealed a normally configured right lung without any cystic lesions (Figure 2D), leading to the decision that surgery was no longer necessary. Subsequently, the infant was transferred to the basic neonatal care unit. There, she received kangaroo care, was breastfed, and was administered a prophylactic vaccination against Respiratory Syncytial Virus (Palivizumab, one dose). On her 30th day of life, the baby was discharged home with supplemental oxygen support.

## Case 2 report

A male newborn from cesarean section at 37 weeks and 1 day of gestation (B.W. 4.140 g) because of severe maternal pre-eclampsia; uterine atony occurred during the procedure. A true knot in the umbilical cord was observed at birth. The patient had severe neonatal asphyxia requiring resuscitation protocol for 5 min and therapeutic hypothermia.

In the neonatal intensive care unit (NICU), both umbilical venous and arterial catheters (UVC, UAC) were placed; PN and conventional IMV were started. Chest x-ray showed good lung volume, normal cardiac axis, well-positioned UVC (T9) and UAC (T6); the abdominal x-ray was unremarkable. A 12-hour video EEG and blood tests for the hypothermia protocol were scheduled. Cooling was maintained at 33.5°C.

On the first day of life, the patient remained stable tolerating hypothermia. Afterward, the baby developed pulmonary hypertension, requiring HFOV, and inhaled nitric oxide (iNO) 20 ppm. Concurrent findings included myocardial ischemia, elevated transaminases, proteinuria, and grade II hemorrhage (brain ultrasound), consistent with multi-organ damage secondary to asphyxia.

The patient ended the hypothermia protocol successfully with normal blood cell counts, coagulation, and hepatic and renal function. Persistent pulmonary hypertension was managed with HFVO and iNO to maintain SaO2 between 90% and 92%. Fasting was maintained and PN was infused solely through the UVC.

**Figure 3:**
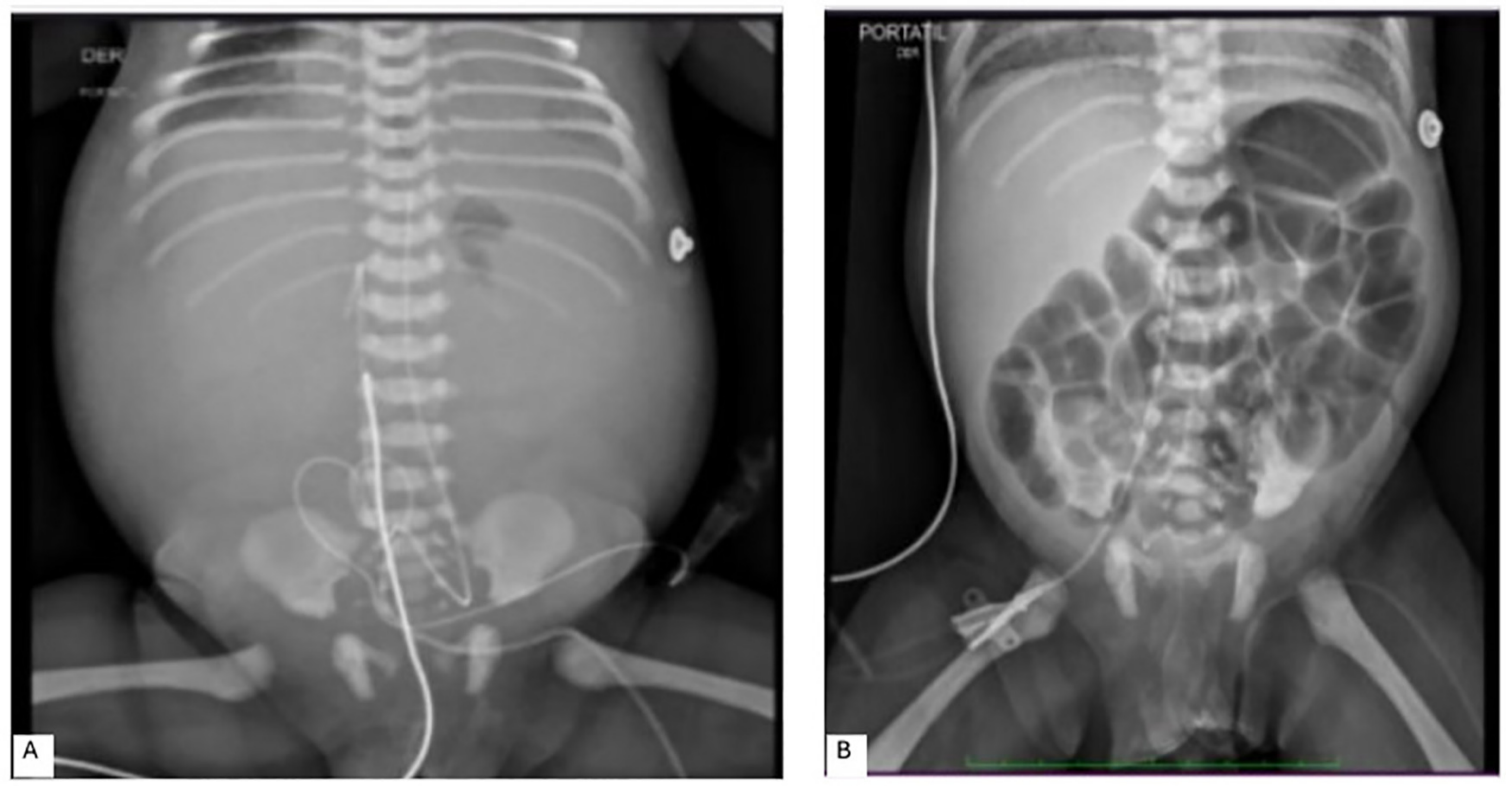
Abdominal x-ray images: **(A)** abundant peritoneal fluid with an umbilical venous catheter showing abnormal angulation. An umbilical arterial catheter is *in situ*. **(B)** Normally arranged intestinal loops without evidence of pneumatosis, with a right femoral catheter present.

On the fourth day of life, the patient began to reveal fast worsening abdominal distension and deteriorated respiratory status leading the O_2_ supply to 100%. Clinical signs suggested fluid accumulation despite laboratory findings excluded secondary ascites or intra-abdominal bleeding. An urgent abdominal x-ray (Figure 3A), revealed ample peritoneal fluid and anomalous angled UVC's position. A paracentesis was performed in the lower right abdominal quadrant, evacuating 260 cc of milky material, consistent with PN extravasation.

Respiratory and abdominal conditions were restored. UVC and UAC were removed, and a femoral CVC was positioned. The patient's improvement was documented by a control x-ray (Figure 3B). Thereafter, the patient's clinical condition gradually improved, and the baby was discharged without the need for therapeutic support, with exclusive breastfeeding.

## Discussion

Central lines are essential in newborns for administering fluids, PN, medications, and hemodynamic controls. The best central line at birth, UVCs, can be in place for a maximum of 5–7 days; the alternatives are Centrally Inserted Central Catheter (CICC) or ECC (7), inserted through a peripheral vein to the superior vena cava. Both catheters are safer long-term options (8).

Neonatologists are increasingly concerned about complications associated with CVCs, including dislocation, infection, thrombosis, emboli, arrhythmia, and organ injury (notably liver injury) (9).

In the first clinical case, the patient presented with hydrothorax, pneumothorax, and pneumatocele following PN extravasation, which resolved after fluid drainage. Notably, lung cavitation's rapid, atypical formation and resolution suggested chemical pneumonia, with cavitations disappearing within 10 days and no respiratory compromise at discharge.

Although pulmonary cysts or cavitations due to PN extravasation have not been reported in the literature (6), similar cystic lesions have been documented in other contexts. For instance, in a term newborn with SARS-CoV-2 infection, cavitations resolved completely on follow-up chest CT after antibiotic therapy (10). On the other hand, while hydrothorax is a well-known complication of PN extravasation, it has not been associated with subsequent cavitation or the formation of cystic lesions (11).

In our case, infectious pneumonia secondary to PN extravasation was initially suspected, leading to comprehensive blood tests, including aerobic and anaerobic cultures and a respiratory pathogen panel—all of which returned negative. The patient also exhibited no symptoms of sepsis or pneumonia, such as fever, lethargy, or decreased appetite. Prophylactic antibiotics were administered based on infectious disease recommendations until blood cultures confirmed negative results.

Another differential diagnosis to consider is congenital lung malformations (CLMs) (12, 13), which result from abnormal airway development during lung morphogenesis, and can lead to cysts or adenomatous areas (14). However, CLMs can be excluded in our patient, as they are typically detected on prenatal ultrasound (US) and confirmed postnatally (15). The first follow-up x-ray showed no focal pulmonary lesions, confirming the prenatal findings.

Our final diagnosis suggests a rare pulmonary complication following pleural PN extravasation, likely secondary to chemical pneumonia and resulting in lung cysts or cavitations that were rapidly resolved with lung tissue recovery.

The second case, by contrast, exemplifies a common complication arising from an indwell UVC: displacement with extravasation of PN into the peritoneal cavity (16). Abdominal conditions improved following the paracentesis and removal of the umbilical catheter, without infections or hepatic sequelae.

We reported this case to underscore that catheter displacement should never be disregarded as a potential etiological factor in these vulnerable patients, who hold multiple pathologies and complications. Therefore, it is imperative to add this possibility into the spectrum of differential diagnoses when facing cases of ascites or acute abdominal presentations in such clinical scenarios (3, 6).

One of the main challenges in managing acute abdomen in preterm infants is distinguishing necrotizing enterocolitis (NEC) from other conditions. Armagan et al., in Z Geburtshilfe Neonatologie, reported a 29-week preterm infant with suspected NEC, presenting with abdominal distension and reduced bowel movements. Further evaluation revealed PN extravasation due to UVC malposition. After catheter removal and drainage, the infant improved, highlighting the need for careful differential diagnosis in cases with nonspecific abdominal signs (17).

In our case, extravasation from a central line also mimicked NEC, causing acute abdominal symptoms. Both these examples stress the importance of considering catheter-related complications. Central lines should be removed as soon as they are no longer needed to reduce the risk of sepsis and complications from displacement or infiltration.

Moreover, UVC malposition in the portal venous system can lead to portal vein thrombosis, hepatic necrosis, and long-term liver damage. To minimize these risks, catheter tip position should be regularly monitored—preferably with USimaging—and lines should be removed once no longer necessary. Routine assessment of liver function may help in the early detection of hepatic complications associated with catheter use (18).

One significant limitation in our experience was the lack of US imaging for regular monitoring of both peripheral and umbilical catheters, which are widely used in neonatal intensive care units nowadays (16, 19, 20). Additionally, the US is crucial for managing pleural effusion and post-drainage surveillance of pneumothorax, as seen in the first clinical case. Unfortunately, sonography is not readily available in our intensive care setting, limiting our ability to implement this monitoring modality (21, 22).

Echography is now commonly used for the placement of CVCs and ECCs due to its greater precision. x-ray determination of the catheter tip's location relies on its projection relative to non-vascular landmarks such as the carina, vertebral bodies, or diaphragm. In contrast, the US directly visualizes the catheter tip within the vasculature, offering superior accuracy (23). As a result, discrepancies between x-rays and echography can be as high as 60% (24).

For these reasons, several studies and guidelines recommend adopting real-time ultrasound as the “gold standard” for confirming correct tip location (23–25). Zini et al. recently emphasized the importance of training neonatal intensive care unit teams to improve catheter management and reduce complications (26). This could be achieved through serial US assessments every 48–72 h (19–21, 27, 28).

## Conclusions

We report two cases of PN extravasation from CVCs leading to life-threatening complications: one involving a rare pleural displacement of the catheter and the other involving a more common displacement of the UVC in a neonatal intensive care unit where only x-ray diagnostic and monitoring support was available. These cases highlight the potentially severe complications associated with UVCs and ECCs, emphasizing the importance of considering these conditions in the differential diagnosis.

Our experience underscores the critical need for ultrasound availability, as exclusive reliance on x-rays in settings without sonography can result in more severe complications and delays in diagnosis and treatment.

## Data Availability

The original contributions presented in the study are included in the article/Supplementary Material, further inquiries can be directed to the corresponding author.
